# Equine encephalosis in Thoroughbred foals on a South African stud farm

**DOI:** 10.4102/ojvr.v82i1.966

**Published:** 2015-09-30

**Authors:** John D. Grewar, Peter N. Thompson, Carina W. Lourens, Alan J. Guthrie

**Affiliations:** 1 Western Cape Department of Agriculture: Veterinary Services, Elsenburg, South Africa; 2 Department of Production Animal Studies, University of Pretoria, South Africa; 3 Equine Research Centre, University of Pretoria, South Africa

## Abstract

Thoroughbred foal body temperature data were collected from shortly after birth until shortly after weaning during the 2007/2008 season on a stud farm in the Western Cape Province of South Africa. Equine encephalosis (EE) caused by EE virus (EEV) serotype 4 (EEV-4) occurred in the foal group during the first autumn after their birth (March and April 2008). A descriptive study was undertaken to provide data on the EEV maternal antibody status, the association between pyrexia and EEV infection, and the incidence of infection amongst the foals prior to and during the episode. This included the frequent capturing of foal body temperature data and regular collection of serum and whole blood during pyretic episodes. Infection by EEV was determined using both virological and serological methods. A high EE incidence of at least 94% occurred amongst the foal cohort, despite the fact that 37% of foals had previously shown maternal antibody to EEV-4. Pyrexia in foals was not directly associated with EE infection and 41% of infected foals showed no detectable pyretic episode. Information obtained from this EE episode showed the high incidence of EEV infection in foals during the first autumn after their birth. Monitoring foal body temperature can alert farmers to outbreaks of infectious disease, such as EE. These results are relevant to the epidemiology of EE and facilitate greater understanding of it as a differential diagnosis of African horse sickness (AHS), given that EE and AHS have similar epidemiologic profiles.

## Introduction

Equine encephalosis (EE) is an arboviral disease of equids that is transmitted by *Culicoides* midges (Paweska & Venter 2004). The causative agent of EE is EE virus (EEV), a member of the *Orbivirus* genus in the *Reoviridae* family. EEV infections of horses are typically unapparent or manifest as only a mild or subclinical disease (Crafford et al. 2011). Clinical signs of EE can include inappetance, pyrexia, mucous membrane congestion and icterus. In some cases, however, the clinical signs may be similar to those seen in cases of African horse sickness (AHS), which is caused by the closely related AHS virus (AHSV) that is also transmitted by haematophagous *Culicoides* midges (Howell, Guthrie & Coetzer 2004). The viraemic period in EEV-infected horses is generally brief, and horses do not act as long-term carriers of the virus (Erasmus et al. 1970). The mortality rate of EE is less than 5% (Howell et al. 2004).

Although the first definitive description of EE in South Africa occurred in 1967 (Erasmus et al. 1970), it is speculated that in 1910, when Theiler described what he called ‘ephemeral fever’ (Theiler 1910), he was describing EE (Guthrie, Pardini & Howell 2009). Seven different serotypes of EEV have been identified in southern Africa, and are assigned as numerical isolates, namely serotype one (Bryanston), serotype two (Cascara), serotype three (Gamil), serotype four (Kaalplaas), serotype five (Kyalami), serotype six (Potchefstroom) and serotype seven (E21/20) (Howell et al. 2002).

EEV is transmitted by a number of different species of *Culicoides* midges and there are EEV-competent vector populations of these midges circulating in the Western Cape Province (Paweska & Venter 2004; Theodoridis et al. 1979; Venter, Koekemoer & Paweska 2006). Horse populations in South Africa have a high EEV seroprevalence, and all seven serotypes of EEV have been prevalent within the Western Cape Province (Howell et al. 2002, 2008; Paweska & Venter 2004).

Whilst there have been prior studies on the seroprevalence of EEV infection of horses in South Africa, this study provides data on the EEV maternal antibody status, the association between pyrexia and EEV infection, and the incidence of infection amongst Thoroughbred foals prior to and during a natural infection with EEV. The major importance of EEV infection of horses in a regional context is its similar epidemiology to that of AHSV infections; however, it is to be stressed that morbidity and mortality of EE is minimal, in marked contrast to those associated with outbreaks of AHS.

## Research method and design

### Body temperature data collection

Temperature data from 127 Thoroughbred foals in the 2007/2008 season were obtained by scanning temperature-sensitive microchips that were inserted into the nuchal ligament of each foal shortly after birth. The microchips used were Destron Fearing™ Lifechip^®^ with Bio-Thermo^®^ technology (Destron Fearing, USA). After being scanned, the microchip returned a string including both the unique identifier of the microchip as well as the body temperature of that foal according to International Organisation for Standardisation (ISO) standard 11785. Microchips were inserted according to manufacturer specifications by a registered veterinarian. Foals were born and resident on a Thoroughbred stud in the Western Cape Province of the Republic of South Africa. A single body temperature measurement was collected from each foal on each weekday from August 2007 until end of July 2008, with recordings beginning at approximately 7 am each day. Recording of each foal’s temperature began on the first weekday morning after that foal was microchipped; 83% of foals had their first body temperature recorded less than 2 days after birth. A total of 34 foals were removed from the study (all for reasons unrelated to EEV infection) prior to the outbreak that began in mid-March 2008; thus the final study cohort consisted of 93 foals. Data collection included a total of 9556 temperature scans from the initial scan on 06 August 2007 up to and including scans on 30 April 2008. The total number of temperature scans during the estimated outbreak period of 15 March 2008–30 April 2008 was 2526 scans.

### Blood and serum collection

Serum samples were collected from each foal by jugular venipuncture at monthly intervals using serum vacuum tubes (Becton Dickenson Vacutainer™, Becton Dickenson, USA) from birth until after the outbreak. At least one serum sample from every foal was selected from each of the pre-outbreak and post-outbreak periods for testing. Whole blood samples for virus isolation (VI) were collected by jugular venipuncture from pyretic foals into heparinised (Becton Dickenson Vacutainer™ 102 IU LH) vacuum tubes, and nine haemolysed samples were excluded from testing.

### Virological and serological methods

Virus isolation was performed as described (Quan et al. 2008) with cytopathic agents identified as EEV by a group-specific antigen capture enzyme linked immunosorbent assay (c-ELISA) (Crafford et al. 2003). EEV isolates were serotyped using a serotype-specific plaque inhibition neutralisation test after the technique described (Porterfield 1960) and modified (Quan et al. 2008), in which electrical insulating fish-spine beads filled with type-specific antiserum were used to indicate homologous virus-antibody neutralisation of virus inoculated Vero cells. Serotype-specific neutralising antibodies to EEV were detected by using a serum neutralisation test (SNT) performed in microtitre plates using EEV-serotype four (EEV-4) as the challenge virus as previously described (Howell et al. 2002).

### Case definition

Pyrexia was determined through statistical process control and was defined as any foal body temperature greater than 39.9 °C or any foal body temperature greater than 1.96 standard deviations above the running mean of the six previous body temperature readings for that individual foal.

Cases of EE that occurred during March and April of 2008 were identified by either or both of the following criteria:

- EEV isolation from whole blood samples collected from a pyretic foal during the outbreak
- seroconversion of foals to EEV, defined as a fourfold or greater increase in antibody titre between serum samples collected prior to 15 March 2008 and after 30 April 2008.

### Statistical methods

Data analysis was performed using chi-squared and Fisher’s exact tests of association using NCSS software (NCSS [2004], Kaysville, Utah, USA). A *p*-value < 0.05 was considered significant. Box plots were produced using R (R [2013], R Foundation for Statistical Computing, Vienna, Austria).

## Results

### Outbreak identification

Analysis of temperatures of foals obtained via the implanted microchips identified a cluster of pyretic episodes between the second half of March and the end of April 2008, with a total of 53/93 (57%) foals having at least one pyretic episode during this period. Prior to this period there was an average of seven pyretic episodes per month from the foal cohort, and after April 2008 this dropped to an average of three episodes per month.

### Pre-outbreak and post-outbreak antibody levels

**FIGURE 1:**
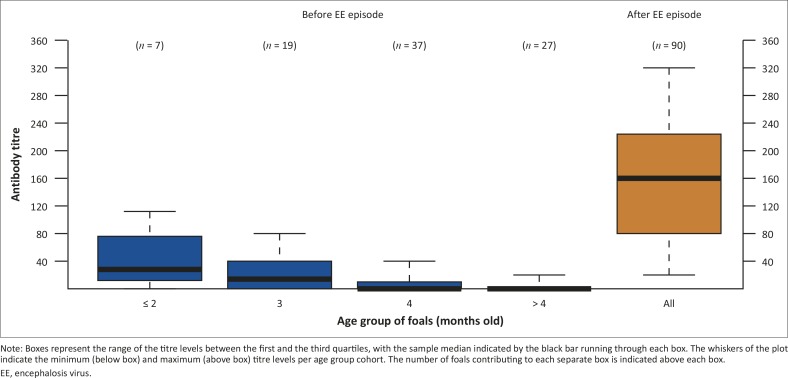
Boxplot of foal age-grouped equine encephalosis virus – serotype 4 antibody titres determined by a serum neutralisation test from the same cohort of foals (*n* = 90) sampled between January and March 2008 (before EE episode – blue box fill) and after 30 April 2008 (after EE episode – orange box fill).

Between January and March 2008, prior to the outbreak of EE, 34/93 (37%) foals had detectable levels of SNT antibodies to EEV-4, the titres of which were waning as foals aged (Figure 1). Based on the titres waning with age and the time of year during which samples were collected, it is assumed that these pre-outbreak titres were maternally derived.

### Test results

Virus isolation was undertaken on blood collected from 44 of the 53 foals that experienced pyretic episodes, and EEV was isolated from 37 (84%) of these foals. The 37 virus isolates were all confirmed to be EEV-4 using the EEV group-specific c-ELISA and serotype-specific plaque inhibition tests. Serum neutralisation test assays confirmed that most foals (90%; *n* = 84) seroconverted to EEV over the outbreak period. Of the nine foals that did not seroconvert to EEV, three foals were not evaluated as they were removed from the study cohort during the outbreak period; six others had equivocal serological responses that did not meet the criteria for seroconversion but were still consistent with possible exposure to EEV. EEV was not isolated from six foals that experienced a pyretic episode and seroconverted to the virus.

In total, 87/93 foals (94%) were considered to have become infected with EEV-4 during the outbreak between mid-March and the end of April 2008. In contrast, only 51 EEV-infected foals (59%) had a pyretic episode during this period. There was no significant association between pyrexia and EEV infection (*p* = 0.3). Re-evaluation of the body temperature data using pyrexia cut-offs at 39 °C and 39.5 °C still showed no significant association between pyrexia and EE infection (*p* > 0.3).

## Ethical considerations

Ethics approval of research was granted by the University of Pretoria’s Animal Use and Care Committee (protocol V075/07). Owner/manager consent was obtained prior to the research and was a criterion of the ethics approval.

## Discussion

We describe the occurrence of EE amongst Thoroughbred foals at a stud in the Western Cape Province of South Africa during March and April 2008. Post-outbreak SNT titre levels were markedly higher than the waning maternal antibody titres prior to the outbreak (Figure 1), illustrating the high incidence of EE outbreak infection and subsequent seroconversion. The high incidence of infection is consistent with the results of other sero-epidemiological studies performed in South Africa, where seroprevalence was found to be 77% (Paweska & Venter 2004) and 84% (Howell et al. 2008). The high incidence amongst the foal cohort precluded determination of any specific risk factors associated with EEV infection of horses on this farm.

EE is described as a generally mild to subclinical infection that in some cases can manifest signs similar to AHS (Howell et al. 2004). Pyrexia monitoring identified the outbreak of EE but only 59% of infected foals exhibited this clinical sign of disease. There were no deaths attributed to EE during the outbreak, which is consistent with the low mortality rate described previously (Howell et al. 2004). Whilst this study concluded that pyrexia in foals is not directly associated with EE, it is evident that if farmers monitor foal body temperature regularly and frequently, an outbreak of EE (and potentially other infectious diseases) can be identified based on an increase in the number of foals presenting with pyrexia within a short period of time. Pyrexia as a result of EEV infection prompting a disease outbreak response has been described during the 2008/2009 EEV outbreak amongst horses in Israel (Aharonson-Raz et al. 2011).

## Conclusion

This study has confirmed that EEV maternal antibody may not prevent EEV infection of foals, most likely because of the serotype-specific nature of maternal antibody (Howell et al. 2008) as well as waning maternal antibody levels prior to the high-risk outbreak season. The fact that at least 34 of 93 foals (37%) had maternal antibodies indicates that their dams were previously exposed to EEV-4. The risk period for arbovirus infection in Thoroughbred foals, particularly those diseases transmitted by *Culicoides* spp*.* like AHS and EE, is in the first autumn following their birth. This susceptibility reflects not only the necessary environmental conditions that provide for peak populations of the insect vector, but also the set management principle in the Thoroughbred breeding industry in the southern hemisphere of having foals born from August through November.
